# Prevalence of Stress, Anxiety, and Depression Among Bangladeshi School‐Going Adolescents: A Cross‐Sectional Study

**DOI:** 10.1002/hsr2.72260

**Published:** 2026-04-08

**Authors:** Md. Shahadat Hossain, Md. Juwel Sheikh, B. M. Abdullah Al Mamun, Md. Shimul Bhuia, Md. Torequl Islam

**Affiliations:** ^1^ Department of Psychology Gopalganj Science and Technology University Gopalganj Bangladesh; ^2^ Department of Pharmacy Gopalganj Science and Technology University Gopalganj Bangladesh; ^3^ Bioinformatics and Drug Innovation Laboratory BioLuster Research Center Ltd. Gopalganj Dhaka Bangladesh

**Keywords:** adolescence, anxiety, depression, gender, grade, stress

## Abstract

**Background:**

Mental health challenges among adolescents are a growing global concern, particularly in low‐ and middle‐income countries such as Bangladesh, where school‐going adolescents often face demanding psychological and academic stressors. These stressors may contribute to the development of mental health issues, including stress, anxiety, and depression.

**Aims:**

In this context, the present study sought to investigate the prevalence of these mental health problems and examine the impact of gender and academic grade levels among Bangladeshi school students.

**Methods:**

A cross‐sectional survey was conducted from July 2024 to March 2025 with a random sample of 400 students from eight government schools in the Gopalganj district. The Bangla version of the Depression Anxiety Stress Scale‐21 (DASS‐21) was used to collect responses from participants.

**Results:**

Results showed a high prevalence with students who experienced stress being 75% (*n* = 300), students with anxiety being 81.3% (*n* = 325), and students with depression being 81.8% (*n* = 327). Female students showed significantly higher levels than males across all three domains (*p* < 0.001). Moreover, as the grade increased, stress, anxiety, and depression increased significantly, with the highest level being in the 10th and 12th grades (board examinations). Mediation analysis showed that anxiety serves as a partial mediator between stress and depression, suggesting that anxiety induced by stressors may lead to depressive symptoms.

**Conclusion:**

The findings underscore the importance of gender‐sensitive and grade‐specific mental health intervention needs among Bangladeshi school students.

## 1 Introduction

Mental disorders account for approximately 16% of the global burden of disease and injury among adolescents, with depression, anxiety, and stress being the most prevalent [1]. These conditions not only affect adolescents current well‐being but can also lead to long‐term psychosocial consequences if left unaddressed [2]. Adolescence, typically defined as the period between 10 and 19 years of age, represents a critical stage of human development marked by rapid and simultaneous physical, psychological, and social changes [1, 3]. These transformations make adolescents especially vulnerable to a range of health issues, including mental health disorders such as depression, anxiety, and stress [4, 5]. The pressure to adapt to sudden bodily changes, cope with increasing academic and societal demands, and navigate complex social relationships often leaves adolescents exposed to emotional distress, especially in developing countries where awareness and mental health services are limited [6, 7].

Existing literature indicates that adolescents in developing countries are increasingly experiencing mental health challenges such as depression, anxiety, and stress. For example, a cross‐sectional study involving 350 students from a boarding school in Malaysia, utilizing the 21‐item depression, anxiety, and stress scale, reported prevalence rates of 39.7% for depression, 67.1% for anxiety, and 44.9% for stress [8]. Similarly, among secondary school students in Manipur, the reported prevalence was 19.5% for depression, 24.4% for anxiety, and 21.1% for stress, whereas much higher rates were found in Chandigarh, with 65.53% for depression, 80.85% for anxiety, and 47.02% for stress [9, 10]. In another study, 41.5% of 545 Saudi Arabian high school females exhibited symptoms of depression, 66.2% showed symptoms of anxiety, and 52.5% experienced stress [11]. Furthermore, a separate investigation involving adolescent school males found that 38.2% had depression, 48.9% experienced anxiety, and 35.5% reported stress [12]. Based on these results, we put forward the following hypothesis:

Hypothesis 1 The prevalence of depression, anxiety, and stress among Bangladeshi adolescents will be comparable to rates reported in other developing countries.

Beyond overall prevalence, gender differences are crucial in adolescents mental health, with previous research showing mixed findings on mental health outcomes. Several studies have reported no significant gender‐based differences in depression, anxiety, and stress among school‐aged adolescents [13, 14, 15]. In contrast, other studies have shown a significantly higher prevalence of depression, anxiety, and stress among female adolescents [9, 10, 16, 17, 18, 19, 20] or males [21, 22]. Given the inconsistent findings in prior research regarding gender differences in adolescents mental health, the present study hypothesizes that:

Hypothesis 2 Female adolescents will report significantly higher levels of depression, anxiety, and stress compared to males.

Besides gender, academic grade levels significantly affects adolescents mental health. Research shows grade 9 students are especially vulnerable due to rising academic demands and concurrent physical and emotional changes, often overlooked in the educational system [23]. In higher grades, the psychological burden intensifies. Grade 10 students frequently encounter heightened stress related to board examination outcomes [24]. Students in grade 11 often struggle with the abrupt shift from secondary to higher secondary school, which requires adjustment to a new academic environment, including unfamiliar teachers, peers, and routines [25]. Meanwhile, grade 12 students face pressure not only from forthcoming board examinations but also from uncertainties regarding their future academic or career choices, contributing to an increased prevalence of depression, anxiety, and stress [25].

Hypothesis 3 Students in higher grades (grades 10–12) will report significantly higher levels of depression, anxiety, and stress compared to grade 9 students.

Numerous studies have identified a positive relationship between anxiety and stress, and that stress arising from poor academic performance, pressure to succeed, exams, assignments, rankings, and teacher‐student dynamics and related factors are key contributors to student anxiety [26, 27, 28, 29]. Anxiety is widely recognized as a natural physiological reaction to stress [30]. Additionally, substantial evidence suggests a strong association between symptoms of anxiety and depression among students [30, 31, 32]. Prolonged exposure to high stress levels, especially when paired with maladaptive coping strategies, may lead students to experience emotional exhaustion, hopelessness, and, in severe cases, the onset of depressive disorders [33, 34]. Drawing on these findings, the following hypothesis is formulated:

Hypothesis 4 Anxiety will act as a mediator between stress and depression in adolescents.

**Figure 1:**
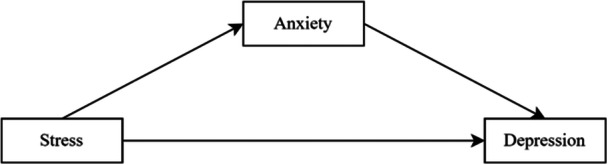
Conceptual model.

The conceptual framework of this study is represented in the Figure 1.

## 2 Methods

### 2.1 Participants

This study utilized a multistage cluster sampling approach. In the initial stage, eight government schools in Gopalganj district were selected via a lottery method. In the subsequent stage, one section per academic grade was randomly selected through a lottery method from each chosen school. In the final stage, student rosters from the selected sections obtained from school records‐were used to randomly draw 50 participants per school without replacement through a lottery method, assigning numbers to students and selecting until the target sample was achieved. A total sample of 400 students (54.8% females and 45.2% males) was selected. Participants ages ranged from 13 to 17 years with a mean age of 15 (SD = 1.41). The distributions of academic grades were 9th grade (28.5%), 10th grade (23.5%), 11th grade (22.2%), and 12th grade (25.8%). Inclusion criteria comprised enrollment in grades 9–12, age 13–17 years, and voluntary participation. Students with diagnosed psychiatric disorders or receiving psychotropic medication were excluded to prevent confounding of self‐reported stress, anxiety, and depression levels.

### 2.2 Study Design

A cross‐sectional survey design was used. Data were collected from July 2024 to March 2025. The Human Resource Ethics Committee, Department of Psychology, Jagannath University (Registration No. HREC/JnU/Psy/21‐2024) granted all procedures.

### 2.3 Measures

The depression anxiety stress scale: The DASS‐21 [35, 36] was used. It contains 21 items rated on a four‐point Likert scale from 0 (did not apply to me at all) to 3 (applied to me very much). These items are grouped into three subscales assessing depression (items 3, 5, 10, 13, 16, 17, 21), anxiety (items 2, 4, 7, 9, 15, 19, 20), and stress (items 1, 6, 8, 11, 12, 14, 18). Subscale scores were summed and multiplied by two. The Bangla DASS‐21 demonstrated good reliability, with Cronbach's alpha of 0.789, 0.799, 0.765, and 0.908 for depression, anxiety, stress, and total score, respectively. To determine the prevalence and severity of symptoms, the following established cut‐off scores for the DASS‐21 were utilized: for stress, scores were categorized as normal (0–14), mild (15–18), moderate (19–25), severe (26–33), and extremely severe (34+); for anxiety, the ranges were normal (0–7), mild (8–9), moderate (10–14), severe (15–19), and extremely severe (20+); and for depression, classifications included normal (0–9), mild (10–13), moderate (14–20), severe (21–27), and extremely severe (28+).

### 2.4 Procedure

After school selection, permission was obtained from the school authorities. For participants under 18, written parental consent and student assent were secured. Participation was voluntary, with the right to withdraw at any time. Data were collected from consenting students during regular class hours, ensuring privacy through adequate spacing. Trained psychology researchers administered self‐report measures—including a personal information form and the DASS‐21 while maintaining strict confidentiality and anonymity.

### 2.5 Statistical Analysis

Statistical analyses were performed using SPSS 27.0. Descriptive statistics (mean, SD, age range) were calculated, and normality (skewness and kurtosis) and homogeneity of variance were assessed (Levene's test). Pearson's correlation examined relationships among stress, anxiety, and depression. Gender differences were analyzed using independent‐samples *t‐*tests, and grade‐level differences with one‐way ANOVA and Bonferroni post hoc tests. Mediation analysis employed PROCESS v4.2 [37]. Analyses were two‐tailed at *α* = 0.01, following SAMPL and Assel et al. [38] guidelines.

## 3 Results

**Figure 2:**
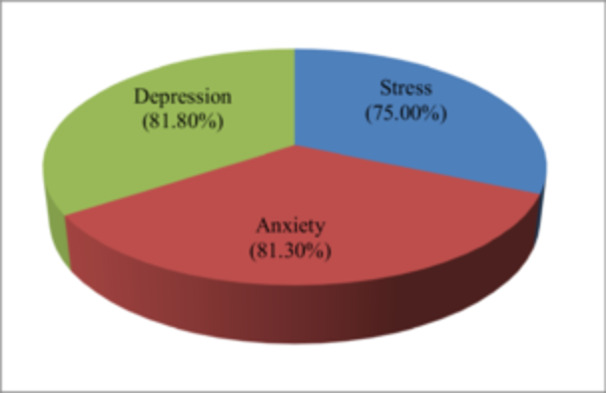
Percentage of adolescents scoring at or above the mild severity level on the DASS‐21 (stress ≥ 15, anxiety ≥ 8, depression ≥ 10). Percentages are based on a total sample of 400 students.

Normality of stress, anxiety, and depression scores was assessed using skewness and kurtosis. Skewness values were 0.05 (stress), 0.46 (anxiety), and 0.32 (depression), and kurtosis values were −0.59 (stress), −0.40 (anxiety), and −0.55 (depression), indicating fairly symmetrical distributions. These results fall within acceptable ranges (skewness ± 0.5; kurtosis ± 2), suggesting that the data approximated a normal distribution [39, 40, 41]. A Pearson‐product moment correlation between all three variables showed a significant positive association. For example, there is a strong correlation between stress and anxiety(*r* = 0.70, *p* < 0.01), stress and depression (*r* = 0.72, *p* < 0.01). There is also a strong positive correlation between anxiety and depression (*r* = 0.71, *p* < 0.01). These results indicate that as stress levels increase, both anxiety and depression are likely to increase as well, and that anxiety and depression are closely related to each other. The data of Figure 2, percentages represent students scoring at or above the mild severity level: stress ≥ 15, anxiety ≥ 8, and depression ≥ 10. It indicated that a significant majority of adolescents experience these psychological challenges. Specifically, 75% (*n* = 300) of the adolescents reported experiencing stress, 81.3% (*n* = 325) indicated experiencing anxiety, and 81.8% (*n* = 327) reported depression. These findings suggest that a majority of students experienced at least some level of emotional difficulty, ranging from mild to extremely severe symptoms.

As can be seen in Table 1, females reported significantly higher levels of stress (*M* = 22.43, SD = 9.55) compared to males (*M* = 17.83, SD = 8.18), *t* = −5.107, *p* < 0.001, 95% CI [−6.36, −2.83], with a medium effect size (Cohen's *d* = 0.56). Similarly, females exhibited higher anxiety levels (*M* = 17.92, SD = 9.71) than males (*M* = 13.56, SD = 8.29), *t* = −4.773, *p* < 0.001, 95% CI [−6.16, −2.56], with a medium effect size (Cohen's *d* = 0.50). Depression levels were also significantly higher for females (*M* = 21.08, SD = 10.09) compared to males (*M* = 14.19, SD = 7.43), *t* = −7.617, *p* < 0.001, 95% CI [−8.65, −5.10], with a large effect size (Cohen's *d* = 0.82).

**Table 1:** Summary of *t*‐test.

Variables	Males		Females		*t*	Cohen's *d*	95% CI for mean difference
	*M*	SD	*M*	SD			
Stress	17.83	8.18	22.43	9.55	−5.107***	0.56	[−6.36, −2.83]
Anxiety	13.56	8.29	17.92	9.71	−4.773***	0.50	[−6.16, −2.56]
Depression	14.19	7.43	21.08	10.09	−7.617***	0.82	[−8.65, −5.10]

*** *p* < 0.001.

Table 2 shows significant grade‐group differences in stress, anxiety, and depression. For stress, significant differences were found (*F* (3, 396) = 87.76, *p* < 0.001) with a large effect size (η² = 0.40). Similarly, anxiety (*F* (3, 396) = 27.11, *p* < 0.001) and depression (*F* (3, 396) = 27.05, *p* < 0.001) also exhibited significant differences, both with large effect sizes (η² = 0.17).

**Table 2:** One‐way ANOVA for stress, anxiety, and depression by grades.

Variables	9th grade (*n* = 114)	10th grade (*n* = 94)	11th grade (*n* = 89)	12th grade (*n* = 103)	*F* (3, 396)	*p*	*η*²
	*M* (SD)	*M* (SD)	*M* (SD)	*M* (SD)			
Stress	12.35 (7.71)	24.26 (5.65)	18.72 (7.52)	27.05 (7.52)	87.76	< 0.001	0.40
Anxiety	10.77 (7.64)	17.17 (8.12)	15.35 (9.10)	21.07 (9.31)	27.11	< 0.001	0.17
Depression	12.47 (8.20)	20.15 (8.54)	17.19 (8.68)	22.72 (9.69)	27.05	< 0.001	0.17

*Note:* Homogeneity of variances tested by Levene's test (*p *> 0.05).

Bonferroni post hoc contrasts (Tables 2 and 3) showed that mean stress was significantly different (*p* < 0.01) between 9th grade (*M* = 12.35, SD = 7.71) and 10th grade (*M* = 24.26, SD = 5.65), and between 9th grade and 11th grade (*M* = 18.72, SD = 7.52), as well as between 9th grade and 12th grade (*M* = 27.05, SD = 7.52). There were also significant differences between 10th and 11th grade, and between 11th and 12th grade. However, the difference between 10th and 12th grades, while significant, showed a smaller mean difference. Similarly, we found significant differences (*p* < 0.01) in mean anxiety levels between 9th grade (*M* = 10.77, SD = 7.64) and 10th grade (*M* = 17.17, SD = 8.12), between 9th grade and 11th grade (*M* = 15.35, SD = 9.10), and between 9th grade and 12th grade (*M* = 21.07, SD = 9.31). There were no significant differences between 10th and 11th grade (*p* > 0.01). The difference between 10th and 12th grade was also significant. For depression, significant differences (*p* < 0.01) were observed between 9th grade (*M* = 12.47, SD = 8.20) and 10th (*M* = 20.15, SD = 8.54), between 9th grade and 11th grade (*M* = 17.19, SD = 8.68), and between 9th grade and 12th grade (*M* = 22.72, SD = 9.69). There were no significant differences between 10th and 11th grades, or between 10th and 12th grade (*p* > 0.01).

**Table 3:** Bonferroni post hoc contrasts between grades for stress, anxiety, and depression.

Variables	Comparison	Mean diff.	SD	*p*	95% CI	
					LL	UL
Stress	9th vs. 10th	−11.90**	1.00	0.001	−14.56	−9.25
	9th vs. 11th	−6.37**	1.02	0.001	−9.06	−3.67
	9th vs. 12th	−14.70**	0.98	0.001	−17.29	−12.11
	10th vs. 11th	5.54**	1.06	0.001	2.72	8.35
	10th vs. 12th	−2.79*	1.02	0.040	−5.51	−0.08
	11th vs. 12th	−8.33**	1.04	0.001	−11.09	−5.57
Anxiety	9th vs. 10th	−6.40**	1.19	0.001	−9.55	−3.24
	9th vs. 11th	−4.58**	1.21	0.001	−7.78	−1.37
	9th vs. 12th	−10.30**	1.16	0.001	−13.37	−7.22
	10th vs. 11th	1.82	1.26	0.899	−1.53	5.17
	10th vs. 12th	−3.90**	1.22	0.009	−7.13	−0.67
	11th vs. 12th	−5.72**	1.24	0.001	−9.00	−2.44
Depression	9th vs. 10th	−7.68**	1.22	0.001	−10.92	−4.43
	9th vs. 11th	−4.72**	1.24	0.001	−8.01	−1.42
	9th vs. 12th	−10.24**	1.19	0.001	−13.41	−7.08
	10th vs. 11th	2.96	1.30	0.140	−0.49	6.40
	10th vs. 12th	−2.57	1.25	0.246	−5.89	0.75
	11th vs. 12th	−5.53**	1.27	0.001	−8.90	−2.15

Abbreviations: 9th = 9th grade, 10th = 10th grade, 11th = 11th grade, 12th = 12th grade, CI = confidence interval, LL = lower limit, UL = upper limit.

* *p* < 0.05

** *p* < 0.01.

Results of Table 4 indicated a significant proportion of variance, with stress accounting for 49% of the variance in anxiety (*R*² = 0.49) and stress and anxiety jointly explaining 60% of the variance in depression (*R*² = 0.60). Specifically, stress had a significant effect on anxiety (path a; *B* = 0.70, *p* < 0.001), and anxiety significantly predicted depression (path b; *B* = 0.42, *p* < 0.001). Additionally, the indirect effect of stress on depression through anxiety was significant (*B* = 0.30, 95% CI [0.22, 0.37]), suggesting that anxiety partially mediates the relationship. The direct effect of stress on depression remained significant but was smaller (*B* = 0.45, *p* < 0.001). The total effect of stress on depression was significant (*B* = 0.75, *p* < 0.001), reinforcing the role of anxiety as a partial mediator in this model.

**Table 4:** Mediation analysis of the model.

Path	*B*	*β*	SE	*t*	*p*	95% CI	
						LL	UL
a: stress → anxiety	0.70	0.70	0.04	19.40	< 0.001	0.63 0.78	
b: anxiety → depression	0.42	0.41	0.05	9.21	< 0.001	0.33 0.51	
c (total): stress → depression	0.75	0.72	0.04	20.49	< 0.001	0.67 0.82	
c′ (direct): stress → depression	0.45	0.43	0.05	9.75	< 0.001	0.36 0.54	
a*b (indirect): stress → anxiety → depression	0.30	0.28	0.04	—	—	0.22 0.37	

Abbreviations: *β* = standardized coefficient, *B* = unstandardized coefficient, CI = confidence interval, LL = lower limit, SE = standard error, UL = upper limit.

## 4 Discussion

The present study aimed to assess the prevalence of depression, anxiety, and stress (DAS) among Bangladeshi school‐going adolescents. Our first hypothesis is that the prevalence of depression, anxiety, and stress among Bangladeshi adolescents will be comparable to rates reported in other developing countries. The findings confirmed this hypothesis, revealing alarmingly high prevalence rates of 75% for stress, 81.30% for anxiety, and 81.80% for depression among the adolescents. These results are notably higher than those reported in comparable studies from other South Asian regions. For instance, a cross‐sectional study conducted in Kathmandu, Nepal, reported DAS prevalence rates of 56.5%, 55.6%, and 32.9%, respectively [25]. Similarly, in an exploratory study among school‐going adolescents in Delhi, India, the prevalence of depression, anxiety, and stress was found to be 47.9%, 65.3%, and 51.8%, respectively [24]. These comparisons suggest that Bangladeshi adolescents experience a significantly higher mental health burden than their regional peers. This discrepancy may be attributed to structural limitations within Bangladesh's mental healthcare system, including a lack of public mental health facilities, a scarcity of skilled mental health professionals, inadequate allocation of financial resources, weak stewardship of mental health policies, and pervasive stigma, all of which collectively render mental healthcare services insufficient and difficult to access for adolescents [42].

Our second hypothesis that female adolescents will report significantly higher levels of depression, anxiety, and stress compared to males. The findings of this study supported the hypothesis, revealing that female students reported significantly higher levels of depressive, anxiety, and stress symptoms compared to their male counterparts. This is consistent with earlier research conducted in Dhaka, Bangladesh, which found a higher prevalence of mental health issues among adolescent females than males [43]. Within the Bangladeshi sociocultural context, female adolescents face unique societal pressures that may further exacerbate psychological distress. These include expectations of early marriage, restricted mobility, greater household responsibilities, and limited autonomy in educational and career‐related decision‐making [44]. Several factors may explain this gender disparity. Females often show more internalizing symptoms, heightened sensitivity to relationships, and hormone‐related conditions (e.g., premenstrual dysphoric disorder), contributing to depression [45, 46]. Pubertal biological changes may trigger anxiety, and increased exposure to psychosocial stressors, including sexual or domestic violence, further elevates vulnerability to anxiety and stress [16, 20].

Our third hypothesis that students in higher grades (grades 10–12) will report significantly higher levels of depression, anxiety, and stress compared to grade 9 students. The study results supported the hypothesis, indicating that stress, anxiety, and depression levels were significantly higher among students in higher grades compared to those in lower grades. Findings revealed significant differences in stress, with 9th graders reporting lower stress compared to 10th, 11th, and 12th graders, mirroring those found in earlier studies [47, 48]. This supports earlier research indicating that academic demands and social pressures increase with grade levels, leading to higher stress [49]. Additionally, significant differences in anxiety levels were observed, with 9th graders reporting lower anxiety compared to 10th, 11th, and 12th graders, in accordance with earlier findings [47, 50]. This reflects the overwhelming nature of transitioning to high school and the associated academic and social challenges [48]. Regarding depression, significant differences were also found, with higher levels of depressive symptoms in 10th, 11th, and 12th graders compared to 9th graders, which aligns with findings documented in a prior study [47]. This pattern may be attributed to the cumulative effect of stress and anxiety on students mental health (depression) as they progress through high school, as well as the added pressure of preparing for post‐secondary education [49]. Findings also showed that stress, anxiety, and depression were higher in 10th and 12th grades compared to 9th and 11th grades. This supports earlier research indicating that on comparison between board grades (10th and 12th) and non‐board grades (9th and 11th), it was observed that depression, anxiety, and stress in board grades were higher in comparison to non‐board grades. This result is compatible with established findings that this could be due to increased tension of approaching board exams of class 10th and 12th, with fear of failure or poor performance in the examinations, which are believed to be a major milestone for future study and career opportunities [51]. Interestingly, despite both being board examination years, grades 10 and 12 did not differ significantly in levels of depression. This finding is consistent with prior evidence suggesting that psychological distress among Bangladeshi adolescents is shaped less by the timing of specific board examinations and more by sustained academic pressure, intense competition, and strong societal and parental expectations across secondary education [17, 52, 53].

Our fourth hypothesis that anxiety will act as a mediator between stress and depression in adolescents, the results of this study support the hypothesis. Finding revealed that stress is significantly associated with depressive symptoms, suggesting that as stress increases, so does the risk of depression. This result is consistent with earlier studies showing a strong link between stress and depressive symptoms [54, 55]. Additionally, anxiety symptoms were found to significantly predict depressive symptoms, aligning with evidence that anxiety and depression often co‐occur, with anxiety frequently preceding the onset of depression [56]. Findings also demonstrated that stress not only directly predicts depressive symptoms but also exerts its effects indirectly through anxiety, confirming a partial mediation [56, 57, 58]. According to the stress response theory, students who face ongoing stress are likely to experience anxiety, which may then lead to depressive outcomes [30].

## 5 Conclusion and Future Directions

The present study assessed the prevalence of stress, anxiety, and depression among school‐attending adolescents in Bangladesh, emphasizing variations by gender and academic grade. A considerable proportion of participants reported symptoms across these three mental health domains. Female students demonstrated elevated levels in all domains relative to males. Psychological distress was more pronounced among those in higher academic grades, particularly grades 10 and 12, presumably owing to pressures from board examinations. Mediation analyses revealed that anxiety partially mediated the stress‐depression relationship, indicating that stress‐elicited anxiety may contribute to depressive symptomatology in this population.

Certain limitations warrant acknowledgment. The cross‐sectional design inhibits causal attributions, and the findings may lack generalizability beyond the eight schools sampled in Gopalganj district. Self‐report measures are susceptible to response biases. Subsequent research ought to incorporate longitudinal or experimental approaches, broader and more diverse samples, and multi‐informant or clinical evaluations. Despite these constraints, the results emphasize the necessity for school‐based mental health interventions in Bangladesh that are attuned to gender and grade‐specific needs.

## Author Contributions

All authors made a significant contribution to the work reported, whether that is in the conception, study design, execution, acquisition of data, analysis, and interpretation, or in all these areas that is, revising or critically reviewing the article; giving final approval of the version to be published; agreeing on the journal to which the article has been submitted; and confirming to be accountable for all aspects of the work. All authors have read and approved the final version of the manuscript.

## Funding

The authors have nothing to report.

## Conflicts of Interest

The authors declare no conflicts of interest.

## Transparency Statement

The corresponding authors, Md. Shahadat Hossain and Md. Shimul Bhuia affirms that this manuscript is an honest, accurate, and transparent account of the study being reported; that no important aspects of the study have been omitted; and that any discrepancies from the study as planned (and, if relevant, registered) have been explained.

## Data Availability

Data available from the authors on request.

## References

[1] World Health Organization , “Adolescent Mental Health,” published 2019, https://www.who.int/news-room/fact-sheets/detail/adolescent-mental-health.

[2] World Health Organization , “Mental Health Status of Adolescents in South‐East Asia: Evidence for Action,” published 2017, https://apps.who.int/iris/handle/10665/254982.

[3] Directorate General of Family Planning , “National Strategy for Adolescent Health 2017–2030,” Ministry of Health and Family Welfare, Government of the People's Republic of Bangladesh, published December 2016, https://www.unicef.org/bangladesh/sites/unicef.org.bangladesh/files/2018-10/National-Strategy-for-Adolescent-Health-2017-2030.pdf.

[4] V. Patel , A. J. Flisher , S. Hetrick , and P. McGorry , “Mental Health of Young People: A Global Public‐Health Challenge,” Lancet 369, no. 9569 (2007): 1302–1313, 10.1016/S0140-6736(07)60368-7. 17434406

[5] World Health Organization , “Adolescent Mental Health,” published July 27, 2021, https://www.who.int/news-room/fact-sheets/detail/adolescent-mental-health.

[6] A. Khalid , F. Qadir , S. W. Y. Chan , and M. Schwannauer , “Adolescents' Mental Health and Well‐Being in Developing Countries: A Cross‐Sectional Survey From Pakistan,” Journal of Mental Health 28 (2019): 389–396, 10.1080/09638237.2018.1521919. 30451053

[7] E. Alslman , N. Abu Baker , H. Dalky , et al., “Mood and Anxiety Disorders Among Adolescent Students in Jordan,” Eastern Mediterranean Health Journal 23, no. 9 (2017): 604–610, 10.26719/2017.23.9.604. 29178117

[8] S. Wahab , F. N. A. Rahman , W. M. H. Wan Hasan , et al., “Stressors in Secondary Boarding School Students: Association With Stress, Anxiety and Depressive Symptoms,” Asia‐Pacific Psychiatry 5, no. S1 (2013): 82–89, 10.1111/appy.12067. 23857842

[9] K. Kumar and B. Akoijam , “Depression, Anxiety and Stress Among Higher Secondary School Students of Imphal, Manipur,” Indian Journal of Community Medicine 42, no. 2 (2017): 94–96, 10.4103/ijcm.IJCM_266_15. 28553025 PMC5427869

[10] R. Sandal , N. Goel , M. Sharma , R. Bakshi , N. Singh , and D. Kumar , “Prevalence of Depression, Anxiety and Stress Among School Going Adolescent in Chandigarh,” Journal of Family Medicine and Primary Care 6, no. 2 (2017): 405–410, 10.4103/2249-4863.219988. PMC5749094 29302555

[11] K. S. Al‐Gelban , H. S. Al‐Amri , and O. A. Mostafa , “Prevalence of Depression, Anxiety and Stress as Measured by the Depression, Anxiety, and Stress Scale (DASS‐42) Among Secondary School Girls in Abha, Saudi Arabia,” Sultan Qaboos University Medical Journal 9, no. 2 (2009): 140–147. 21509290 PMC3074779

[12] K. S. Al‐Gelban , “Depression, Anxiety and Stress Among Saudi Adolescent School Boys,” Journal of the Royal Society for the Promotion of Health 127, no. 1 (2007): 33–37, 10.1177/1466424007070492. 17319315

[13] S. K. Bhasin , R. Sharma , and N. K. Saini , “Depression, Anxiety and Stress Among Adolescent Students Belonging to Affluent Families: A School‐Based Study,” Indian Journal of Pediatrics 77 (2010): 161–165, 10.1007/s12098-009-0260-5. 19936655

[14] N. Manikandan and S. Nirmala Devi , “A Study on Stress Among Adolescent Learners,” Scholarly Research Journal for Interdisciplinary Studies 2, no. 16 (2015): 2725–2730, http://oaji.net/articles/2015/1174-1426244624.pdf.

[15] B. Shaikh , P. Doke , and J. Gothankar , “Depression, Anxiety, Stress, and Stressors Among Rural Adolescents Studying in Pune and a Rural Block of Nanded District of Maharashtra, India,” Indian Journal of Public Health 62, no. 4 (2018): 311–314, 10.4103/ijph.IJPH_174_17. 30539897

[16] D. M. Christiansen , “Examining sex and gender differences in anxiety disorders,” in A fresh look at anxiety disorders, ed. F. Durbano (InTechOpen, 2015), 17–49, ISBN: 978‐953‐51‐2149‐7. 10.5772/60662.

[17] M. S. Islam , M. E. Rahman , M. S. Moonajilin , and J. van Os , “Prevalence of Depression, Anxiety and Associated Factors Among School Going Adolescents in Bangladesh: Findings From a Cross‐Sectional Study,” PLoS One 16 (2021): e0247898, 10.1371/journal.pone.0247898. 33793610 PMC8016317

[18] M. K. Mridha , M. M. Hossain , M. S. A. Khan , et al., “Prevalence and Associated Factors of Depression Among Adolescent Boys and Girls in Bangladesh: Findings From a Nationwide Survey,” BMJ Open 11 (2021): e038954, 10.1136/bmjopen-2020-038954. PMC7813352 33455924

[19] B. Preeti , K. Singh , and R. Kumar , “Study of Depression, Anxiety and Stress Among School Going Adolescents,” Indian Journal of Psychiatric Social Work 8, no. 1 (2017): 6–9.

[20] L. E. Reardon , E. W. Leen‐Feldner , C. Hayward , et al., “A Critical Review of the Empirical Literature on the Relation Between Anxiety and Puberty,” Clinical Psychology Review 29 (2009): 1–23, 10.1016/j.cpr.2008.09.005. 19019513 PMC2652567

[21] X. Jiang , H. Zheng , R. Yang , S. Wang , and H. Zhong , “Retrospective Analysis of Clinical Characteristics and Treatment of Children and Adolescents With Depression,” Frontiers in Psychiatry 14 (2023): 1036314, 10.3389/fpsyt.2023.1036314. 37575578 PMC10412874

[22] C. D. Deb , P. Chatterjee , K. Walsh , et al., “Anxiety Among High School Students in India: Comparisons Across Gender, School Type, Social Strata, and Perceptions of Quality Time With Parents,” Australian Journal of Educational and Developmental Psychology 10, no. 1 (2010): 18–31, https://eprints.qut.edu.au/33012/.

[23] M. N. Sarif , V. Vandana , et al., “Stress, Anxiety, and Depression Among School Students: A Cross‐Sectional Study,” Journal of Indian Education 48, no. 3 (2022): 135–150.

[24] A. Kumar , G. Yadav , N. Chauhan , and S. Bodat , “Prevalence of Depression, Anxiety, and Stress Among School‐Going Adolescents in Delhi: A Cross‐Sectional Study,” International Journal of Community Medicine and Public Health 6 (2019): 5021–5026, 10.18203/2394-6040.ijcmph20195177.

[25] A. Karki , B. Thapa , P. M. S. Pradhan , and P. Basel , “Depression, Anxiety and Stress Among High School Students: A Cross‐Sectional Study in an Urban Municipality of Kathmandu, Nepal,” PLOS Global Public Health 2, no. 5 (2022): e0000516, 10.1371/journal.pgph.0000516. 36962418 PMC10022099

[26] M. Fawzy and S. A. Hamed , “Prevalence of Psychological Stress, Depression and Anxiety Among Medical Students in Egypt,” Psychiatry Research 255 (2017): 186–194, 10.1016/j.psychres.2017.05.027. 28575777

[27] E. Venkatarao , S. Iqbal , and S. Gupta , “Stress, Anxiety & Depression Among Medical Undergraduate Students & Their Socio‐Demographic Correlates,” Indian Journal of Medical Research 141 (2015): 354–357, 10.4103/0971-5916.156571. 25963497 PMC4442334

[28] N. M. Kouzma and G. A. Kennedy , “Self‐Reported Sources of Stress in Senior High School Students,” Psychological Reports 94 (2004): 314–316, 10.2466/pr0.94.1.314-316. 15077784

[29] G. S. M. Leung , K. C. Yeung , and D. F. K. Wong , “Academic Stressors and Anxiety in Children: The Role of Paternal Support,” Journal of Child and Family Studies 19, no. 1 (2010): 90–100, 10.1007/s10826-009-9288-4.

[30] R. Dyson and K. Renk , “Freshmen Adaptation to University Life: Depressive Symptoms, Stress, and Coping,” Journal of Clinical Psychology 62, no. 10 (2006): 1231–1244, 10.1002/jclp.20295. 16810671

[31] N. Azad , A. Shahid , N. Abbas , A. Shaheen , and N. Munir , “Anxiety and Depression in Medical Students of a Private Medical College,” Journal of Ayub Medical College, Abbottabad: JAMC 29, no. 1 (2017): 123–127, PMID: 28712190. 28712190

[32] R. Shao , P. He , B. Ling , et al., “Prevalence of Depression and Anxiety and Correlations Between Depression, Anxiety, Family Functioning, Social Support and Coping Styles Among Chinese Medical Students,” BMC Psychology 8, no. 1 (2020): 38, 10.1186/s40359-020-00402-8. 32321593 PMC7178943

[33] O. Coskun , A. O. Ocalan , C. B. Ocbe , H. O. Semiz , and I. Budakoglu , “Depression and Hopelessness in Pre‐Clinical Medical Students,” Clinical Teacher 16, no. 4 (2019): 345–351, 10.1111/tct.13073. 31397111

[34] P. Iliceto , M. Pompili , D. Lester , et al., “Relationship Between Temperament, Depression, Anxiety, and Hopelessness in Adolescents: A Structural Equation Model,” Depression Research and Treatment 2011 (2011): 160175, 10.1155/2011/160175. 21789278 PMC3140782

[35] S. A. H. M. Alim , S. M. E. Kibria , M. J. Lslam , et al., “Translation of DASS‐21 into Bangla and Validation Among Medical Students,” Bangladesh Journal of Psychiatry 28, no. 2 (2017): 67–70.

[36] S. H. Lovibond and P. F. Lovibond , Manual for the Depression Anxiety Stress Scales, 2nd ed. (Psychology Foundation of Australia, 1995).

[37] A. F. Hayes , Introduction to Mediation, Moderation, and Conditional Process Analysis: A Regression‐Based Approach, 2nd ed. (The Guilford Press, 2018).

[38] M. Assel , D. Sjoberg , A. Elders , et al., “Guidelines for Reporting of Statistics for Clinical Research in Urology,” Journal of Urology 201, no. 3 (2019): 595–604, 10.1097/JU.0000000000000001. 30633111 PMC6600813

[39] M. G. Bulmer , Principles of Statistics (Dover Publications, 1979).

[40] A. Field , Discovering Statistics Using SPSS, 3rd ed. (Sage Publications Ltd., 2009).

[41] D. George , SPSS for Windows Step by Step: A Simple Guide and Reference (Pearson Education India, 2011).

[42] M. T. Hasan , T. Anwar , E. Christopher , et al., “The Current State of Mental Healthcare in Bangladesh: Part 1— An Updated Country Profile,” BJPsych International 18, no. 4 (November 2021): 78–82, 10.1192/bji.2021.41. 34747942 PMC8554893

[43] N. Islam , N. A. Khan , G. U. Ahsan , et al., “Gender Disparities in Mental Health Status Among the School‐Going Adolescents of Dhaka, Bangladesh,” Bangladesh Journal of Medical Science 16, no. 2 (2010): 151–158.

[44] M. Hasan and M. Al Amin , “Determinants of Depression Among Ever‐Married Adolescent Girls in Bangladesh: Evidence From the Bangladesh Adolescent Health and Wellbeing Survey 2019–2020,” PLoS One 19, no. 11 (2024): e0314283, 10.1371/journal.pone.0314283. 39585812 PMC11588215

[45] C. Kuehner , “Why Is Depression More Common Among Women Than Among Men?,” The Lancet Psychiatry 4 (2017): 146–158, 10.1016/S2215-0366(16)30263-2. 27856392

[46] P. R. Albert , “Why Is Depression More Prevalent in Women?,” Journal of Psychiatry and Neuroscience 40, no. 4 (2015): 219–221, 10.1503/jpn.150205. 26107348 PMC4478054

[47] K. Shamsuddin , F. Fadzil , W. S. W. Ismail , et al., “Correlates of Depression, Anxiety and Stress Among Malaysian University Students,” Asian Journal of Psychiatry 6, no. 4 (2013): 318–323, 10.1016/j.ajp.2013.01.014. 23810140

[48] W. Yuan , “Analysis of Psychological Stress Sources of High School Students and Relevant Countermeasures,” Advances in Social Science, Education and Humanities Research 299 (2019): 620–624, 10.2991/assehr.k.191206.079.

[49] K. Schraml , A. Perski , G. Grossi , and I. Makower , “Chronic Stress and Its Consequences on Subsequent Academic Achievement Among Adolescents,” Journal of Educational and Developmental Psychology 2, no. 1 (2012): 69, 10.5539/jedp.v2n1p69.

[50] T. Xu and H. Wang , “High Prevalence of Anxiety, Depression, and Stress Among Remote Learning Students During the COVID‐19 Pandemic: Evidence From a Meta‐Analysis,” Frontiers in Psychology 13 (2023): 1103925, 10.3389/fpsyg.2022.1103925. 36704682 PMC9871576

[51] S. S. Salelkar and S. Borker , “Prevalence of Depression, Anxiety and Stress Among School Going Adolescents and Their Relationship to Socioeconomic Status,” Indian Journal of Youth and Adolescent Health 7, no. 4 (2020): 8–14, https://pdfs.semanticscholar.org/4075/85b775e96e558f2053b06275983e6b368d41.pdf.

[52] M. R. A. Rabby , M. S. Islam , M. T. Orthy , A. T. Jami , and M. T. Hasan , “Depression Symptoms, Anxiety, and Stress Among Undergraduate Entrance Admission Seeking Students in Bangladesh: A Cross‐Sectional Study,” Frontiers in Public Health 11 (2023): 1136557, 10.3389/fpubh.2023.1136557. 37181689 PMC10169692

[53] M. A. B. Siddik , M. N. Hasan , A. Mahmud , et al., “Prevalence of Depression and Its Associated Factors Among Undergraduate Admission Candidates in Bangladesh: A Nation‐Wide Cross‐Sectional Study,” PLoS One 18, no. 11 (2023): e0295143, 10.1371/journal.pone.0295143. 38033102 PMC10688886

[54] E. M. C. Bouma , J. Ormel , F. C. Verhulst , and A. J. Oldehinkel , “Stressful Life Events and Depressive Problems in Early Adolescent Boys and Girls: The Influence of Parental Depression, Temperament and Family Environment,” Journal of Affective Disorders 105, no. 1–3 (2008): 185–193, 10.1016/j.jad.2007.05.007. 17574686

[55] B. Sharma and R. Wavare , “Academic Stress Due to Depression Among Medical and Paramedical Students in an Indian Medical College: Health Initiatives Cross Sectional Study,” Journal of Health Science 3, no. 5 (2013): 29–38.

[56] C. Zhang , L. Shi , T. Tian , et al., “Associations Between Academic Stress and Depressive Symptoms Mediated by Anxiety Symptoms and Hopelessness Among Chinese College Students,” Psychology Research and Behavior Management 15 (2022): 547–556, 10.2147/PRBM.S353778. 35282002 PMC8906854

[57] N. Falsafi , “A Randomized Controlled Trial of Mindfulness Versus Yoga: Effects on Depression and/or Anxiety in College Students,” Journal of the American Psychiatric Nurses Association 22 (2016): 483–497, 10.1177/1078390316663307. 27566622

[58] V. Lemay , J. Hoolahan , A. Buchanan , et al., “Impact of a Yoga and Meditation Intervention on Students' Stress and Anxiety Levels,” American Journal of Pharmaceutical Education 83, no. 5 (2019): 7001, 10.5688/ajpe7001. 31333265 PMC6630857

